# Design and Production of Bispecific Antibodies

**DOI:** 10.3390/antib8030043

**Published:** 2019-08-02

**Authors:** Qiong Wang, Yiqun Chen, Jaeyoung Park, Xiao Liu, Yifeng Hu, Tiexin Wang, Kevin McFarland, Michael J. Betenbaugh

**Affiliations:** Department of Chemical and Biomolecular Engineering, Johns Hopkins University, Baltimore, MD 21218, USA

**Keywords:** single-chain variable fragment (scFv), bispecific antibody, quadroma technology, knobs-into-holes, CrossMAb, bispecific T-cell engager (BiTE)

## Abstract

With the current biotherapeutic market dominated by antibody molecules, bispecific antibodies represent a key component of the next-generation of antibody therapy. Bispecific antibodies can target two different antigens at the same time, such as simultaneously binding tumor cell receptors and recruiting cytotoxic immune cells. Structural diversity has been fast-growing in the bispecific antibody field, creating a plethora of novel bispecific antibody scaffolds, which provide great functional variety. Two common formats of bispecific antibodies on the market are the single-chain variable fragment (scFv)-based (no Fc fragment) antibody and the full-length IgG-like asymmetric antibody. Unlike the conventional monoclonal antibodies, great production challenges with respect to the quantity, quality, and stability of bispecific antibodies have hampered their wider clinical application and acceptance. In this review, we focus on these two major bispecific types and describe recent advances in the design, production, and quality of these molecules, which will enable this important class of biologics to reach their therapeutic potential.

## 1. Introduction

Over recent decades, immunotherapies, including checkpoint inhibitors, adoptive cell transfer, monoclonal antibodies, and vaccine treatments, have become efficient and highly specific treatments to fight cancer by boosting a patient’s immune system. These treatments can specifically target tumor cells and the tumor microenvironment with less cytotoxicity and fewer side effects [1]. Through three decades of development and exploration, therapeutic monoclonal antibodies have become the most widely used and approved immunotherapy method in clinical practice to treat various malignant tumors [1]. These antibodies are designed to bind to specific targets found on cancer cells and destroy them by activating the patient’s immune system. More recently, bispecific antibodies represent a valuable alternative antibody platform in immunotherapy treatment. These bispecifics work by binding to two different antigen sites and can provide more robust and tailored immunogenic targeting than what is possible with natural antibodies.

**Figure 1:**
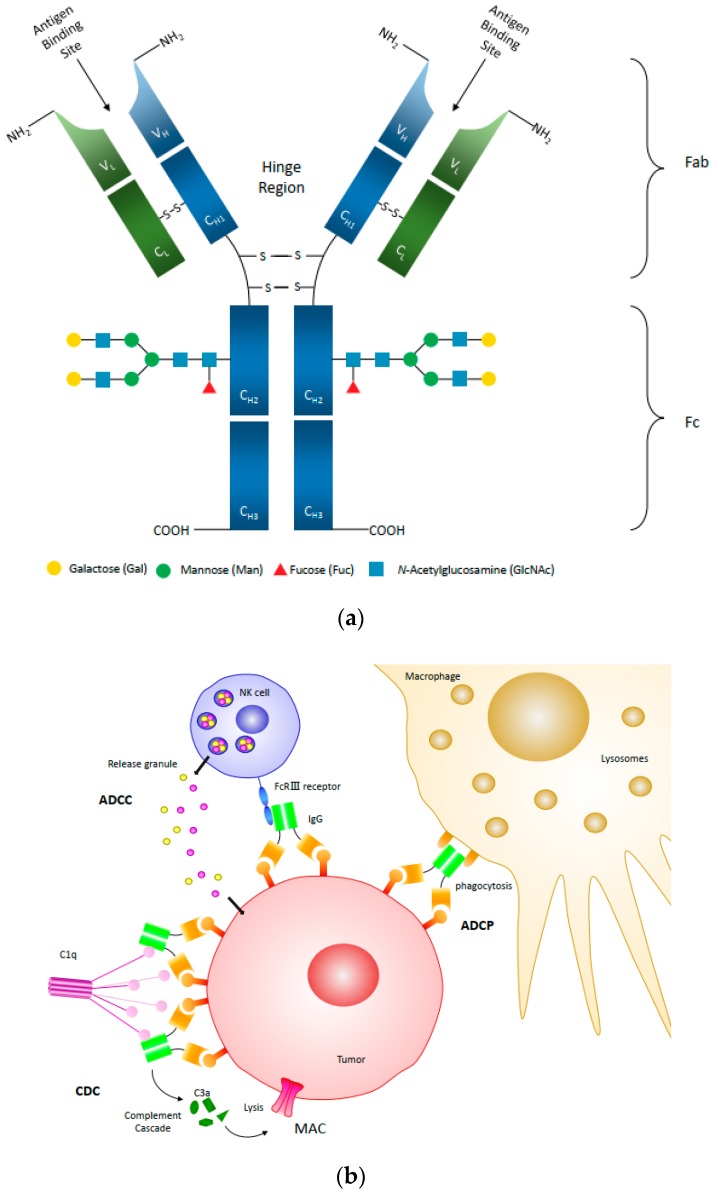
(**a**) The illustration of human IgG_1_ structure with N-glycan attached at Asn297 site in the CH2 of the Fc region. Light chains (L) are highlighted in green, and heavy chains (H) are highlighted in blue. C: constant domain; V: variable domain; H: heavy chain; L: light chain; S-S: disulfide bond; Fab: Fragment antigen-binding domain; Fc: Fragment crystallizable domain. Fc regions which bind effector molecules and cells. (**b**) The schematic diagram of antibody-dependent cellular cytotoxicity (ADCC), complement-dependent cytotoxicity (CDC) and antibody-dependent cell-mediated phagocytosis (ADCP) mechanisms in cancer treatment. For ADCC, natural killer (NK) cells recognize the Fc region mediated by surface FcγRIIIa receptors, causing apoptosis of the antibody-coated tumor cells. Once activated, NK cells release cytotoxic granules containing perforin and granzymes to induce apoptosis in targeted cells [8]. Another antibody-induced pathway is the classical pathway in the complement system, often called complement-dependent cytotoxicity. CDC is initiated when the C1q complex interacts with antibodies bound to the pathogen surface, facilitating the lysis of cells by forming the membrane attack complex (MAC) which induces lethal colloid-osmotic swelling [9,10,11]. The third major effector mechanism is ADCP. ADCP is a potent mechanism by which IgG-opsonized tumor cells activate the FcγRIIa and FcγRI expressed on the surface of macrophages to induce phagocytosis, resulting in engulfment and degradation of the target cell through acidification of the phagosome and the fusion with lysosomes [12].

An antibody produced against a single epitope of an antigen is called a monoclonal antibody (mAb) produced by a single plasma cell type, while polyclonal antibodies bind to multiple epitopes of an antigen or multiple antigens and are typically produced by multiple plasma cells [2]. Bispecific antibodies are engineered artificial antibodies capable of recognizing two epitopes of an antigen or two antigens. Human immunoglobulin G (IgG) is the most common type of antibody found in human serum and is further broken down into four subclasses, IgG_1-4_. These subclasses differ in their constant regions, particularly the ɤ-chain sequences and disulfide bond patterns, but share the same basic structure [3]. As illustrated in Figure 1a, the basic structure of IgG is composed of two light chains (LC) and two heavy chains (HC) to form a complex quaternary Y-shaped structure with three independent protein moieties connected through a flexible hinge region [4]. These moieties are symmetrical with two identical fragment antigen-specific binding (Fab) regions and one fragment crystallizable (Fc) region [3]. Antibodies bind to specific antigens through the Fab domain formed by hypervariable regions of heavy and light chains. Natural Abs can bind to natural and artificial antigens with high affinity and specificity with a remarkable diversity of 10^8^–10^10^ different variants for each antigen-binding site [5,6]. The Fc region of the antibody binds to receptors or other proteins of the host immune system such as Fcγ receptors (FcγRs), C1q, and neonatal Fc receptor (FcRn) to initiate distinct effector functions. Small differences in the amino acid sequence and glycosylation pattern on the Fc domain can highly impact key attributes such as IgG thermal stability, FcγR-binding effectiveness, and serum half-life [7].

Cancer cells are abnormal cells differentiated from normal healthy cells, but they may be difficult for the body’s immune system to detect. Monoclonal antibodies have been successful as cancer therapeutics by targeting surface antigens over-expressed or expressed uniquely, on tumor cells [13]. The efficacy of antibody-based cancer immunotherapy involves two main factors as discussed below: (1) Blocking or binding factors activate cell death or inhibit activation of signal pathways used by cancer cells to grow and survive, resulting in tumor cell death [14,15]. For example, trastuzumab targets HER2 receptors in breast and stomach cancer cells in order to inhibit their proliferation and survival [16], and cetuximab inhibits epidermal growth factor receptor (EGFR) in colorectal and lung cancer [17,18]; and (2) Immune effector functions that engage the Fc region of antibodies via Fc receptors (FcR) on immune cells. The mechanisms of antibody-dependent cellular cytotoxicity (ADCC), complement-dependent cytotoxicity (CDC) and antibody-dependent cellular phagocytosis (ADCP) are illustrated in Figure 1b. All three mechanisms (ADCC, CDC, and ADCP) can induce target cell death and aid in the efficacy of the treatment.

Even with the success of various anti-tumor therapeutic antibody drugs, however, there also have been significant limits to conventional antibody therapy. One major drawback of antibody therapy is low tumor penetration and retention rate. Many therapeutic mAbs directed against tumor-specific antigens largely remain in the circulation with typically only 20% of the administered dose interacting with the surface proteins of solid tumors [19]. This disproportionality reflects the challenges of achieving effective penetration and retention within solid tumor tissue [19]. Furthermore, most mAbs serve to prevent the binding of growth factors to their receptors but fail to induce apoptosis of tumor due to an insufficient immune response from patients, especially the re-activation of T-cells to destroy tumors. For the effector functions (mainly ADCC, CDC, and ADCP) triggered by mAbs, the exact effectiveness depends on specific antibodies. Numerous studies have demonstrated that the amino acid sequence of the CH2 and CH3 domain and the Fc conserved glycan profile both impact the antibody ADCC and CDC activities [11]. Moreover, the unsatisfactory performance of natural antibody treatment also comes from the extensive cross-talk among some signaling pathways in cancer cells and nearby cells contributing to relapses in mAb treatment, which works by blocking signaling pathways and the induction of apoptosis [20,21].

Bispecific antibodies were proposed three decades ago and have been extensively investigated to overcome the limitation of natural mAbs, which can only bind a single epitope [22]. Bispecific antibodies can target two different antigens at the same time [5], such as simultaneously binding tumor cell receptors and recruiting cytotoxic immune cells. This enhanced functionality may potentially result in fewer side effects and fewer injections. Furthermore, from a biopharmaceutical manufacturer’s perspective, fewer clinical trials and reduced production costs can be accomplished by making a single molecule instead of two [23]. With more than 100 bispecifics in clinical trials [5], bispecific antibodies are under development to cover a broad spectrum of applications including diagnosis, imaging, prophylaxis, and therapy, with the majority of drug candidates focusing on cancer therapy [22].

Through decades of exploration and development of bispecific antibodies and their derivatives, there are two common formats of bispecific antibodies on the market: the single-chain variable fragment (scFv)-based (no Fc fragment) antibody and the full-length IgG-based antibody. Unlike the conventional monoclonal antibodies, great production challenges with respect to the quantity, quality, and stability of bispecific antibodies have hampered their wider clinical application and acceptance [24]. Meanwhile, advanced design strategies around phage display screening, antibody linker engineering, quadroma technology [25], knobs-into-holes technology [26], common light chain [27], CrossMAb technology [28], and protein engineering have all been extensively investigated, and make up the principal knowledge base of this fast-growing and diverse field [22,29,30,31]. Therefore, this review will focus on the design and manufacture of these two major bispecific molecule types and describe the recent developments in the therapeutic potential and opportunities in bispecific antibody production capacity and quality achieved by employing a range of operational strategies.

## 2. Strategies to Improve Bispecific Antibody Production and Quality

### 2.1. Single-Chain Variable Fragment (scFv) Antibodies

Single-chain variable fragments (scFvs) are minimalist forms of a functional antibody, generated by fusing variable domains of the IgG heavy chain (VH) and light chain (VL) through a flexible polypeptide linker [32]. ScFv molecules have a molecular weight in the range of 25 kDa, with a single antigen-binding site that is comprised of components from each arm of the antibody [22]. Several important considerations in developing scFv antibodies are the antibody fragment types, the linker type, and production capability. More recently, another exciting area for using scFv technology is the chimeric antigen receptor (CAR) T-cell approach for adoptive cell transfer immunotherapy [33]. Given the scope and volume of this review, the use of scFvs for CAR-Ts will not be included here [34].

#### 2.1.1. Antibody Fragment Types

**Figure 2:**
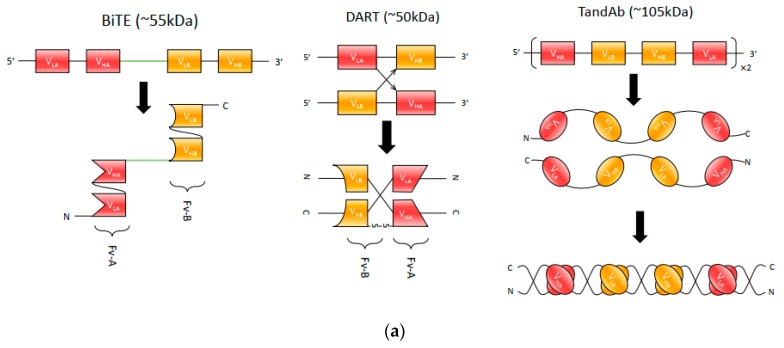

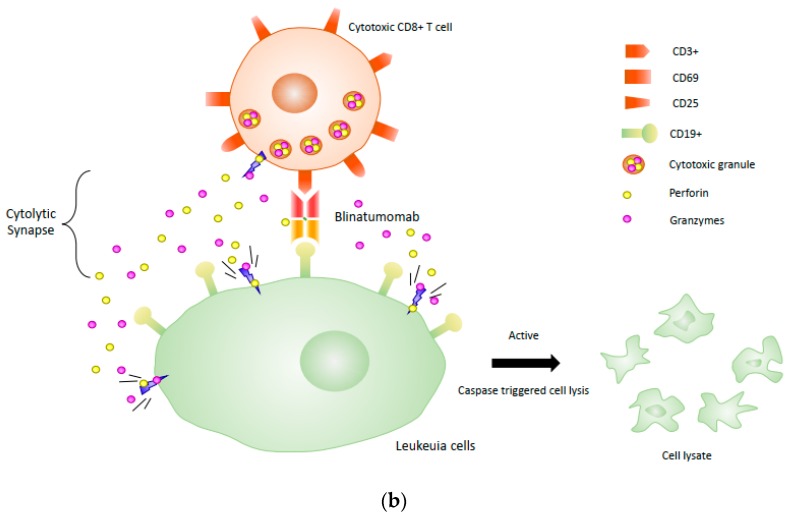
(**a**) The construction of three main bispecific antibody fragment molecules. (**b**) The mechanism of Blinatumomab treatment. Blinatumomab as a bispecific antibody can simultaneously bind to CD3+ T-cells and CD19+ leukemia cells and has been approved for the treatment of B-cell precursor acute lymphoblastic leukemia (ALL).

Currently, there are three main bispecific antibody fragment formats: bispecific T-cell engager (BiTE), dual-affinity re-targeting proteins (DARTs) and Tandem diabodies (TandAbs), as depicted in Figure 2a.

BiTE molecules have been extensively applied in cancer immunotherapy for re-targeting of T-cells to tumor cells or tumor-associated cells in the tumor microenvironment. They employ scFv fragments from two different monoclonal antibodies connected by a peptide linker, enabling them to retain each antibody’s binding activity when assembled [35]. The short flexible linker connecting the two scFvs enables free rotation of the two arms, which is vital for flexible interaction with targeted receptors on two opposing cell membranes (cytotoxic T-cell and tumor cell) and the subsequent induction of T-cell activation [36]. 

One of the most successful BiTE drugs is blinatumomab (Blincyto^®^, DrugBank entry DB09052), which has been approved by the FDA for the treatment of B-cell precursor acute lymphoblastic leukemia (ALL). Blinatumomab is comprised of an anti-CD 19 scFv in the VL-VH orientation linked through a short glycine/serine (GGGGS) linker to an anti-CD3 scFv in the VH-VL orientation [30]. The mechanism of blinatumomab has been illustrated in Figure 2b. Due to its small size, blinatumomab can reach in close proximity to T-cell and target cell membranes, but this feature also leads to the rapid clearance from circulation with a short elimination half-life (mean ± SD) of 1.25 ± 0.63 h [37], which is presumed to be eliminated renally [38]. As a result, BiTE requires continuous dosing at a high concentration (15–28 µg per day) to recruit and activate a large amount of suboptimal T cells to achieve half-maximal target cell lysis [36]. Therefore, this antibody is administered as a 4-week continuous intravenous (IV) infusion to maintain sufficient therapeutic serum concentration [37], which increases costs by having to produce more clinical-grade antibodies [39]. The single polypeptide chain structure that enhances BiTE antibody-antigen recognition, however, comes at the cost of increased aggregation and decreasing protein stability [24,40].

Partly in response to these issues, researchers developed a potential alternative—dual-affinity re-targeting proteins (DARTs). As shown in Figure 2a, a DART is composed of two Fv fragments, with two unique antigen-binding sites formed when two Fv fragments heterodimerize [41]. Specifically, Fv1 consists of a VH from antibody A and a VL from antibody B, while Fv2 is made from a VH from antibody B and VL from antibody A. Unlike BiTE antibodies which are connected by a polypeptide linker, this combination allows DART to mimic natural interaction within an IgG molecule. Adding another cysteine residue to the end of each heavy-chain improves stability by forming a C-terminal disulfide bridge (see Figure 2a) [41]. Compared to a BiTE, DART molecules are able to retain potency for both in vitro and in vivo administration as well but can be produced at scale with lower aggregation rates [21,42]. A recent comparison by Moore et al. of the in vitro ability of CD19xCD3 DART and BiTE molecules to kill B-cell lymphoma found that DART molecules outperformed BiTE molecules consistently. In this study, both DART and BiTE molecules were derived from the same parental antibodies (mouse anti-human CD3 and CD19 mAbs), with DART molecules performing better in maximal B-cell lysis, requiring less concentration for half-maximal B-cell lysis, and in molecular markers of T-cell activation [40].

Currently, DART and BiTE proteins can also be further engineered to integrate better with patient immune systems. For example, several BiTE molecules can be linked to the IgG Fc domain to generate BiTE-Fc fusion drugs compatible with once-weekly dosing for treatments [43,44,45]. Anti-CD19x CD3 BiTE-Fc fusion protein binds with high affinity to human and non-human primate (NHP) CD19 as well as CD3 with a serum half-life of 210 h following a single intravenous administration of a 5 µg/kg dose in NHP, without obvious signs of toxicity in clinical and laboratory animal studies [45]. An anti-BCMA BiTE-Fc fusion protein for the treatment of multiple myeloma has a serum half-life of 112 h following a single 15 µg/kg dose in NHP [44]. In addition, BiTE molecule can also be linked to human albumin to extend serum half-life. A comparison of anti-CD33 x CD3 BiTE-Fc and BiTE-albumin fusion proteins reveals that the Fc-based BiTE antibody constructs provided a similar survival advantage when administered every four or five days as the canonical BiTE when administered on a daily basis in the mouse model. Alternatively, the albumin fusion-based BiTE was less efficacious when administered every four days than the daily administered canonical BiTE [43]. DART proteins can also be fused with the Fc region of an IgG, creating a DART-Fc construct which can significantly extend the serum half-life when compared to the DART protein alone [46,47]. One group designed HIVxCD3 DART and DART-Fc and evaluated their killing activity on mononuclear cell cultures isolated from HIV-infected participants [46]. Their results showed that both DART formats reduced cell-to-cell virus spreading in resting or activated CD4 T-cell cultures [46]. Additionally, though HIVxCD3 DART-Fc performed similarly in killing activity to HIVxCD3 DART, the DART-Fc extended the DART in vivo half-life from less than 10 to 70.2 h [46].

The small size of scFvs contributes to a high renal clearance rate in comparison to natural antibodies. One solution to the size issue is to generate Tandem diabodies (TandAbs), which are shown in detail in Figure 2a. These tetravalent bispecific antibodies provide two binding sites for each antigen to maintain the avidity of a natural bivalent antibody [48,49]. Moreover, TandAbs have a molecular weight (approximately 105 kDa) exceeding the first-pass renal clearance threshold, thus offering a longer half-life compared to smaller antibody constructs [48,50]. Two TandAb format drugs are in clinical trials—AFM13 (CD30xCD16) for NK cell recruitment and AFM11 (CD19xCD3) for T-cell recruitment [51].

#### 2.1.2. Linker Engineering

The linker region between light and heavy-chain domains plays a significant role in stabilizing the antibody and is therefore an important target for optimizing scFvs [52]. The scFv can be assembled with a single polypeptide chain in the form of VH-linker-VL or VL-linker-VH, where the linker bridges the gap between C and N termini of the respective domains. Studies investigating the orientation of the heavy and light-chain domains imply that both orientations can be favorable in different cases and that linker design may impact biophysical properties [53,54,55]. Two essential considerations in linker design are amino acid composition and sequence length. Firstly, the amino acid composition is critical in designing a viable and flexible linker peptide; for instance, a hydrophilic sequence is indispensable to avoid intercalation of the peptide within or between the V domains during protein folding [56]. Currently, the most commonly used amino acid sequence motif is (G4S)_n_ (G: glycine, S: serine; G4S is four glycine residues and one serine residue). Glycine and serine are preferred because their short side chains grant conformational flexibility and minimal immunogenicity, while serine additionally improves solubility [31,57]. Besides the conventional Gly-Ser linker, other designs include charged residues such as glutamic acid and lysine to enhance solubility [58], while high-throughput selection methods such as phage display also facilitate the design and generation of linkers that are specifically optimized for certain antibodies [59].

In addition to composition, the length of the linker between heavy and light chains of the Fv domain is also critical in assembling the correct conformation of the scFv. It has been reported that the linker should be able to span 3.5 nm (35 Å) between the C terminus of one V domain and the N-terminus of the other V domain without affecting the native Fv conformation [31]. The length of the linker exerts a significant impact on multimer formation of the antibodies, with studies showing that a linker length longer than 12 amino acid residues allows sufficient distance between heavy and light-chain domains to associate and form monomers [60,61,62]. Shorter linkers connecting VH and VL can prevent the direct association of the two domains, resulting in an increased possibility for pairings between heavy and light chains of different scFv molecules, forming dimers, trimers or higher order oligomers [40,63]. Therefore, by properly designing the linker length, one can effectively promote the formation of scFv molecules that are designed as diabodies (in particular, the TandAbs molecule), or whose multivalent forms are desired over their monovalent forms. For example, pharmacokinetic studies suggest that for particular antibodies, such as CC49, the formation of dimer or tetramers are favorable, improving tumor targeting compared to the monomeric form [64,65], while maintaining efficient in vivo tumor localization and in blood [66]. Therefore, it is critical to design linker length to achieve desired scFv conformation or distribution of multivalent forms.

The design of linkers for optimal bispecific scFv configurations necessitates careful consideration of the above principles. Initial attempts to construct bispecific scFvs focused on designing linkers that directly connects two monovalent single-chain antibodies. Examples of these designs include the linker CBH1 composed of 24 amino acids [35] and the 205C linker which has 25 amino acid residues [67]. Factors such as the amino acid composition of these inter-chain linkers can have an impact on the function of single-chain bispecific antibodies [68]. More recent designs of linkers emphasize more on linker length and achieving the desired antibody conformations and domain associations, which can be exemplified by BiTE, DART, and TandAb, as shown in Figure 2a. For successful construction of bispecific scFv, it is important to control linker length to avoid or minimize non-cognate pairing between heavy and light chains of heterologous antibodies, since appropriate VH-VL association is critical in antibody affinity and specificity that ensures proper functioning [29,69]. The BiTE design places long linkers between heavy and light chains of homologous domains to ensure association, and short linkers between heterologous heavy-chain fragments (the GGGGS linker) to form the connection between the two Fvs [70]. The DART molecules are dual chains that bind to each other to form functional dimers, where the linkers between VHA and VLB or VHB and VLA needs to be as short as five amino acids to prevent undesired non-homologous pairing [40]. Moreover, the positioning of the disulfide bond is another key feature of DART molecules, which holds the molecule together in the correct orientation. The linker design for TandAb is similar to DART, for which the linkers between adjacent domains are six amino acids (GGSGGS) so that two identical chains are likely to bind and form large dimers that are favorable in the aspects of in vivo half-life [21,48,71].

#### 2.1.3. Stability Engineering of scFv Antibodies

Stability of scFv molecule is a critical factor because it is believed that there is a direct correlation between the stability and biological activity [72,73], and stable scFv molecules can be considered as building blocks for functional bispecific antibodies. A number of different approaches through changing expression environment and introducing helper molecules (e.g., chaperons) to improve the scFv solubility and stability are discussed in the following bispecific scFv expression and production (Section 2.1.4). In this section, we focus on the approaches that achieve optimized protein stability through direct modification of scFv frameworks. Two types of commonly used methods to engineer scFv structure include loop grafting and mutagenesis. The loop grafting approach may be favorable for the generation of therapeutic scFv because the process achieves both stabilization and humanization in one step, by grafting the antigen-specific complementarity determining regions (CDRs) onto frameworks with suitable biophysical properties including stability [74,75,76,77,78]. For example, Borras et al. [79] reported a successful attempt to humanize and stabilize rabbit variable domains by grafting CDRs from 15 different rabbit monoclonal antibodies onto a human scFv scaffold, resulting in similar affinity but significantly improved biophysical properties. Alternatively, for the mutagenesis approaches, enhanced stability is achieved by either optimizing structure by rational site-specific mutation [80,81,82,83,84] or directed protein evolution (i.e., inducing random mutagenesis followed by positive selection steps) [74,85,86,87,88,89,90,91]. Compared to the laborious directed evolution method which requires iterative steps to reach an optimum, site-specific mutagenesis approaches are relatively easy to implement with the well-established techniques [84]. Rational designs of site-specific mutation are generally knowledge-based and different mutations can be combined and introduced simultaneously with the assumption that mutations have cumulative effects on improving stability [84]. For example, one of the most common mutation-based optimization methods is the consensus sequence approach, in which the most frequent amino acid at any position in homologous Fv domain is assumed to contribute to the stability considering molecular evolution and selection, and a mutation toward this collective consensus sequence is expected to have a positive effect on stability [77,81,82]. Besides the consensus approach, other methods alter amino acid residues to achieve certain goals such as creation of inter-domain disulfide bonds [84,92,93,94], creation of intramolecular hydrogen bonds [83,95] and optimization of hydrophobicity [77,83,96]. As a successful case of utilizing these mutation-based approaches, Miller et al. [72] used a combination of sequence-based statistical analyses (residue frequency analysis and consensus methods) and structure-based design approach to identify the target residues for mutations in VH and VL sequences. Then, the scFv mutants were screened through a high-throughput antigen-binding assay with thermal challenges [72]. The isolated stability-engineered scFv variant was able to produce in suspension Chinese hamster ovary cells with high yield (21.5 mg/L), purity and biological activity [72].

#### 2.1.4. Bispecific scFv Antibody Expression and Production

Appropriate host platforms are determinant to the efficient expression and production of scFv antibodies, and there exist several different viable platforms for scFv expression including bacteria, yeast, mammalian cells, insect cells, plant and cell-free systems [97,98,99,100]. Given that bispecific scFvs are composed of two or more scFv molecules, the various expression hosts for the bispecific scFvs may vary from those used for the production of scFv single molecules. The “best” expression system for bispecific scFv proteins is yet to be determined because differences in size, amino acid sequence, and conformation of the recombinant protein make it difficult to conclude a universal expression system that optimizes the yield and quality of the protein, which can be affected by many factors such as solubility and stability [99]. However, several studies listed in Table 1 have reported successful expression of bispecific scFv and its fusion molecules using bacterial and mammalian systems.

*E. coli* is one of the most widely used hosts for scFv expression. Some of the major advantages of using *E. coli* include its rapid growth, cost efficiency, high heterologous protein productivity, well-understood genetics as well as easy genetic manipulation [111,112,113]. Unlike the glycosylated whole antibody protein, scFv molecules are much easier to produce in bacteria. However, challenges still remain for harnessing this high-yield expression system, one of which is insufficient protein solubility. It was reported by multiple studies that proteins produced from the *E. coli* expression system result in misfolding and inclusion body [114,115,116,117]. This inefficiency in producing soluble scFv is known to be caused by the lack of chaperon and post-translational machinery and the reducing environment of *E. coli* cytoplasm which prevents disulfide bonds to be formed [97,118], and for scFv molecules, formation of intra-domain disulfide bonds is essential for the key structure known as the “immunoglobulin fold” [119,120]. Therefore, successful expression of functional scFv molecules from *E. coli* systems usually requires additional procedures or modifications. For example, subsequent protein refolding and recovery steps can be integrated into the process, including solubilization treatment with agents such as urea and guanidine hydrochloride, and a step to refold solubilized protein by removing solubilization agents by methods such as dialysis [121]. Gruber et al. (Table 1, [67]) reported the production of bispecific scFv in *E. coli* with these refolding steps. The solubility of scFv molecules can also be improved by secreting them into the bacterial periplasm that has an oxidizing environment, through genetically attaching the secretory signal sequence to the N-terminus of scFv sequence [7,122,123,124]. A number of studies have reported the periplasmic expression of BiTE type molecules in *E. coli* [4,103,104]. Besides the above methods to tackle solubility issue, there are other approaches exist that may in the future be applied to facilitate the production of bispecific scFvs in bacterial platforms. For example, expression of scFv as a fusion protein with solubility enhancing tags such as MBP, NusA, and TRx can promote and facilitate correct protein folding [38,97,125,126], despite that these tags need to be removed afterward to allow normal antibody usage. Furthermore, studies have shown that co-expression of molecular chaperons such as Skp, OmpH, HlpA and FkpA, and folding modulators/catalysts such as disulfide bond metabolizing enzymes can effectively tackle protein aggregation and misfolding problems, and the “cocktails” approach has been an increasingly common expression strategy that involves the simultaneous usage of various chaperons or folding catalysts [32,127,128,129,130].

Single-chain Fv molecules are also expressed through other platforms to exploit particular advantages that are not granted by bacterial hosts. Mammalian cells represent the most widely used production platform for therapeutic proteins and a promising expression vehicle for bispecific scFvs due to their advanced protein folding pathways and post-translational modifications [99,131]. Mammalian cells allow for stable expression and robust production of soluble recombinant proteins. For example, Vendel et al. [114] and Jain et al. [132] managed to express bioactive scFv molecules via Chinese Hamster Ovary (CHO) cells while the same proteins expressed in bacteria shows less activity or even a significantly different secondary structure.

Besides bacterial and mammalian cells, other expression systems have demonstrated advantages in various studies involving the expression of scFvs and may be potential candidates of large-scale production platforms for bispecific scFvs in the future. Yeast as a eukaryotic microorganism is not only capable of producing correctly folded and fully functional proteins, but can also survive and grow rapidly in simple media [99]. Another organism of interest is insect cell, which allows the utilization of the baculovirus-mediated gene expression system [99]. The advantage of using baculovirus expression vector system (BEVS) is the high gene expression level achieved though the polyhedron gene (polh) promoter in virus-infected insect cells [96,133]. Production of recombinant protein from plant is believed to be a desired method for large-scale protein production considering factors including scalability, cost efficiency, and safety [134]. Growing transiently expressed or stable transgenic plants followed by protein extraction from leaf tissue allows the production of scFv molecules [4,135,136]. Besides the host-based expression systems, cell-free protein synthesis system allows high-throughput protein library generation due to the efficiency and flexibility this approach offers [137,138], and the expression of scFv molecules can be achieved without the time-consuming steps of expression vector generation and transformation.

### 2.2. Full-Size IgG-like Asymmetric Bispecific Antibody

Although IgG-like asymmetric bispecific antibodies have some properties that are similar to natural monoclonal antibodies, they are engineered molecules that have not been generated by typical B-cells [139]. As a result, these differences lead to significant production challenges. One of the greatest challenges for asymmetric IgG-like bispecific antibodies manufacturing is ensuring the correct assembly of antibody fragments, which is a prerequisite for bispecific antibody large-scale production. Random assembly of four distinctive polypeptide chains (two different heavy and two different light chains) results in 16 combinations (10 different molecular configurations), among which only two represent the desirable asymmetric heterodimeric bispecific antibody (12.5% of the statistical probability) [22,140,141]. The remaining pairings result in non-functional or monospecific molecules [22]. So not only the quality but also the quantity of bispecific antibodies generated from *E. coli* and mammalian cells can be greatly improved by optimizing the correct assembly of bispecific antibodies. Production examples of several IgG-like bispecific antibody molecules are summarized in Table 2. There are mainly two problems that must be solved to produce the desired IgG-like bispecific antibody—the heterodimerization of two different heavy chains and the discrimination between the two light-chain/heavy-chain interactions [142]. Judicious genetic and cellular engineering strategies, such as quadroma technology, knobs-into-holes, common heavy chain, and common light-chain strategies, CrossMab and co-culture methods, have been implemented to produce optimized Y-shape IgG-like bispecific antibodies. We will describe each of these important strategies briefly in the following sections. 

#### 2.2.1. Quadroma (or Hybrid-Hybridoma) Technology

**Figure 3:**
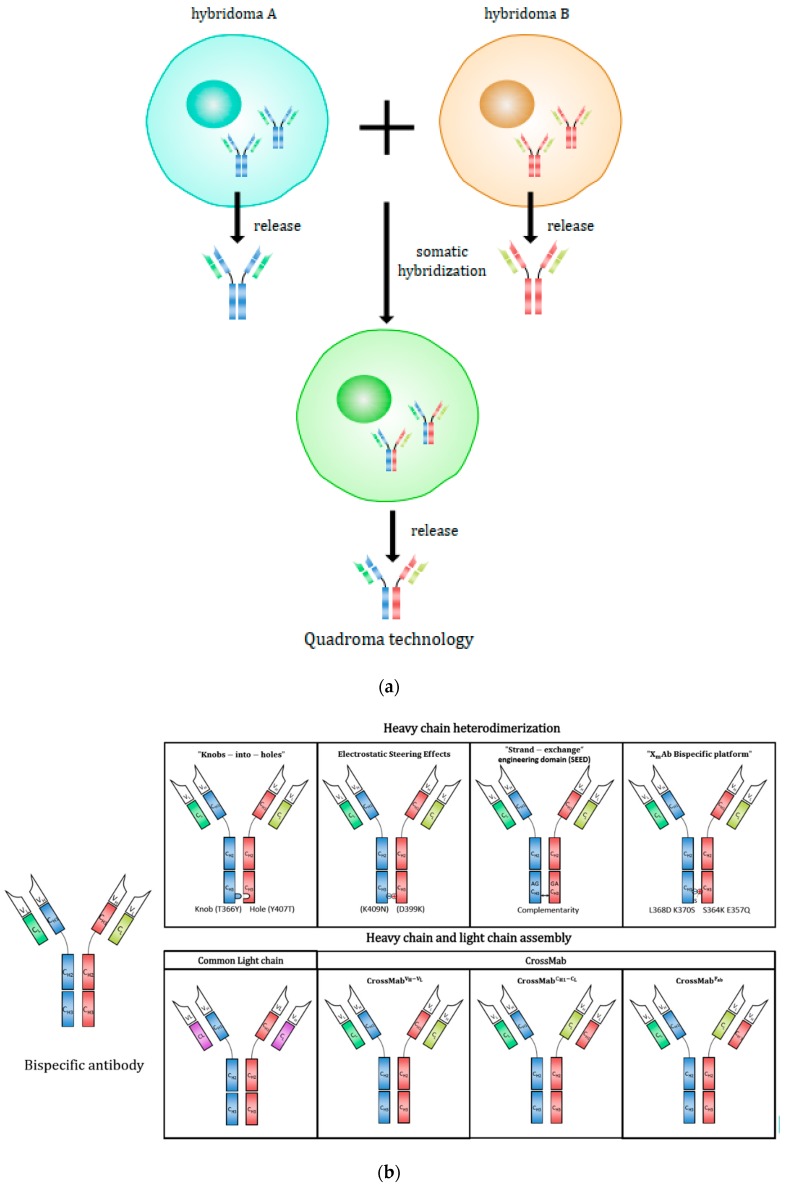
The strategies for improving IgG-like bispecific antibody product quality. (**a**) The illustration of quadroma technology. (**b**) A summary of the heavy and light-chain genetic and protein engineering strategies to achieve homogeneous asymmetric heterodimeric bispecific antibody product.

Initially, a bispecific antibody was generated by the somatic fusion of two hybridomas, as illustrated in Figure 3a. [149]. Each hybridoma cell expresses a unique monoclonal antibody with predefined specificity. Then, the two antibody-expressing cells are fused and the resulting hybrid-hybridoma cell expresses the immunoglobulin heavy and light chains from both parents [149], where assembly allows the formation of both parental and hybrid immunoglobulins. The quadroma technology represents the foundation of bispecific antibody production, but also suffers from low production yields and high product heterogeneity. [4]. The random assembly of two different heavy and two different light chains can theoretically result in 10 different molecular configurations and only one of those is functional bispecific antibody [22]. The real percentage of functional bispecific antibody by a quadroma cell line is unpredictable and a laborious process is required to isolate the bispecific antibody from the side products [150,151]. Later, a chimeric quadroma technology was developed by fusing a murine and a rat hybridoma cell line [139]. The content of chimeric mouse/rat bsAb was significantly enriched due to preferential species-restricted heavy/light-chain pairing in contrast to the random pairing in conventional mouse/mouse or rat/rat quadromas [152,153]. Furthermore, rat heavy chains did not bind to protein A for purification, while the mouse heavy chains in bsAbs can be eluted at pH 5.8 while the full-size parental mouse Ab can be eluted at pH 3.5 [153]. This feature provided an easy and simple purification process through protein A and ion-exchange chromatography to isolate the desired bispecific component. With the improvements of quadroma technology, Catumaxomab (anti-EpCAM x anti-CD3) was the first approved IgG-like bispecific antibody in Europe in 2009 for the intraperitoneal treatment of patients with malignant ascites [154]. Catumaxomab is generated via quadroma technology and composed of mouse IgG2a and rat IgG2b [154]. As a trifunctional antibody, one Fab antigen-binding site binds T-cells via CD3 receptor, the other site binds tumor cells via the tumor antigen epithelial cell adhesion molecule (EpCAM) and the Fc region provides a third binding site to recruit and activate immune effector cells via binding to FcγRI, IIa and III receptors [154]. Nevertheless, Catumaxomab cannot bind to the inhibitory Fcγ IIb receptor. Immunogenicity is another concern—human anti-mouse or anti-rat antibody response are sometimes observed in patients with catumaxomab treatment [25,51].

#### 2.2.2. Heavy-Chain Assembly

Fc heterodimerization is a particularly important design to reduce the number of possible combinations of different forms while exclusively producing asymmetric antibodies by eliminating the formation of normal monoclonal antibodies. Heterodimeric heavy chains are achieved by combining two complementary but not identical heavy chains that result in a single heavy-chain combination. Each heavy chain can then bind to different light chains, resulting in four possibilities: one bispecific molecule, one non-functional combination, and two monospecific molecules [22]. Using this approach, the possible antibody combinations are thus substantially reduced from 10 different molecules to just the four remaining molecules [22]. The dimerization of Fc is achieved by CH3 domain of Fc (the last domain of the constant region) interfacing with each other [155]. Different technologies can be applied to engineer the CH3 domain so that two different Fc domains can be properly linked to one another, as shown in Figure 3b.

Knobs-into-holes technology, which involves engineering CH3 domains to create either a “knob” or a “hole” in each heavy chain to promote Fc heterodimerization [143], has been extensively applied for Fc engineering. The knobs-into-holes model was first proposed by Francis Crick to pack amino acid backbones of coiled-coil alpha-helix domains of proteins [156]. Ridgway et al. applied the knobs-into-holes as a novel design strategy to engineer heavy chains of Fc domains rendering them able to form heterodimers [26]. A small amino acid in a CH3 domain was replaced with larger one (T366Y) to make a knob variant, and a large amino acid in another CH3 domain was replaced with smaller one (Y407T) to produce a hole so that the two engineered domains can fit into one another favoring the heterodimerization [26]. Furthermore, additional mutation sites including S354C and T366W in a CH3 domain were found to generate knobs while Y349C, T366S, L368A, and Y407V were examined in the other CH3 domain for holes while L351C was used to form disulfide bonds and further enhance the heterodimerization [157,158]. The engineering sites were identified and examined according to three criteria: (1) The distances between alpha-carbons should be around 5.0–6.8 Å, which is the average distance found in naturally formed disulfide bonds, but can reach up to 7.6 Å; (2) the pairings of amino acid residues should be distinct from those on each natural CH3 interface; (3) the formation of disulfide bonds between cysteine residues should be favorable conformationally [158]. As a result, the heavy chain (HC) heterodimerization was further improved up to 95% under co-expression conditions, making it feasible for scalable production [143,159]. The knobs-into-holes heterodimerization not only solves the heavy-chain problem via the correct heterodimeric pairing of bispecific antibodies but also renders them conformationally stable [160] and allows for antibody purification by protein A [22]. Zhang et al. demonstrated the stability of knobs-into-holes heterodimers in comparison to holes-holes homodimer variants, further supporting the knobs-into-holes heterodimerization as a rational design strategy [160]. The heteromeric heavy chains produced functional bispecific antibodies and also retained Fc-mediated effector functions, such as ADCC. Compared to an *E. coli* host system producing unglycosylated antibodies, a mammalian host expression systems can produce glycosylated, effector-function competent heterodimeric antibodies. One study revealed that afucosylation of half the asymmetric anti-CD20 antibody by knobs-into-holes technology from CHO cells is sufficient to produce ADCC-enhancement similar to that observed for a fully afucosylated symmetric wild-type anti-CD20 antibody [161].

Alternatively, strand-exchange engineered domain (SEED) heterodimerization represents another steric mutation-based design strategy which utilizes complementarity of alternating sequences derived from IgG and IgA CH3 domains also known as AG SEED CH3 and GA SEED CH3. The IgG and IgA CH3 derivatives generate complementary sequences so that the two complementary heavy-chain heterodimers are assembled while excluding the assembly of homodimers lacking complementarity [162]. According to Muda et al., Fab-SEED fusions retained desirable binding affinity and characteristics comparable to other antibodies including favorable pharmacokinetics and stability [163]. 

In addition to the steric mutations mentioned earlier, electrostatic steering interactions have also been widely used to promote the formation of heavy-chain heterodimers by substituting a residue in a CH3 domain and another residue in the other CH3 domain by a negatively charged aspartic acid or glutamic acid residue and a positively charged lysine residue respectively. Then, the charge pair substitution favors the assembly of heterodimers while inhibiting the formation of homodimers via electrostatic repulsion. Gunasekaran et al. first demonstrated the Fc heterodimerization of antibodies using the electrostatic steering effects as applied to the production of bispecific antibodies [144]. In their work, novel engineering strategy was applied to support favorable opposite charge interactions between heterodimers and also to induce unfavorable repulsive charge interactions between homodimers at the same time by replacing K409 and D399 in different CH3 domains with aspartate and lysine, respectively, in order to suppress the formation of homodimers [144]. 

Indeed, site-specific mutations can significantly improve both the quality and quantity of bispecific antibodies by circumventing the heavy-chain problem. However, the steric mutations and the introduction of charge pairs can reduce the thermostability of bispecific antibodies. Moore et al. reported an efficient method called XmAb bispecific platform, which leads to enhanced thermostability that combines charge interactions, conformational aspects of CH3 domains, and hydrogen bonding [159]. The novel Fc mutations include a side chain swap of native IgG1 to S364K and K370S heterodimer to form a hydrogen bond in between followed by L368D/K370S substitutions to drive salt bridge interactions. The engineered Fc sites were specified and selected based on the minimum exposure area in order to ensure near net-isovolumetric substitutions without interfering the receptor binding or generating extra potential N-linked glycosylation sites [159]. Additionally, due to the engineered structure and charge pair mutations, the formation of homodimers was disfavored by driving the steric hindrance and charge repulsion between the sites [159]. In addition, the sites were examined and engineered to modulate different isoelectric points (pI) between the two CH3 sites. This is of a particular interest to improve the robustness of the heterodimeric Fc platform at scale because an engineered pI of heterodimers significantly different from those of homodimers can facilitate and ease the purification process of heterodimeric bispecific antibodies from non-bispecific antibodies via standard ion-exchange chromatography, whose performance is independent of the variable domains and format of bispecific antibodies.

In summary, bispecific antibody heavy-chain heterodimerization, especially within the CH3 region, represents a rapidly emerging approach including multiple design strategies, such as steric mutation, electrostatic steering interactions and charge difference of heavy chains to facilitate purification. These approaches are often applied together to achieve bispecific antibody heavy-chain heterodimerization with minimum homodimer formation. However, an alternative strategy is to generate a common heavy chain with one lambda and one kappa light chains without any modification, which is called a kappa-lambda (κλ) body [164]. The co-expression of a heavy chain and one κ and one λ in CHO or HEK293 cells generated both monospecific and bispecific antibodies [140,164]. It has been reported that the expression of light chains is a determinant for the bispecific antibody specificity and affinity [164]. Columns specific for kappa- and lambda-monospecific antibodies isolation were then adopted followed by Protein A purification, although only around 50% of the final product is κλ body with the rest mainly including kappa-kappa and lambda-lambda antibody side products [140,164]. Interestingly, another research group reported that codon de-optimization of the lambda chain sequence increased the κλ body yield two-fold and enhanced the relative distribution of bispecific antibodies in a low kappa chain expressing κλ body cell line [165].

#### 2.2.3. Heavy Chain and Light-Chain Assembly

While deliberate modifications of Fc CH3 domains enable correct heavy-chain heterodimerization, using two different light chains still results in a low yield of desired bispecific antibodies (the generation of four different combinations, with only one being bispecific). Advanced approaches have, therefore, been developed to allow the correct pairing of light chain and heavy chain to resolve the improper heavy chain and light-chain interaction problem, such as the common light-chain method and CrossMab to swap the VH and VL Fab fragments partially. These strategies are often applied in combination with Fc-modified heavy chains, as shown in Figure 3b.

First, a common light-chain strategy was applied to assemble IgG-like bispecific antibodies which can be combined together with the knobs-into-holes approach [158]. The mechanism of a common light-chain strategy is based on the fact that antibodies discovered from phase display screening against diverse antigens often share the same VL domain, reflecting the very limited size of the L chain repertoire in the phage library [22]. One of the great advantages of the common light-chain format is that it allows the use of methods that simplify the antibody engineering and the purification process in industrial production [166]. For example, based on computational prediction, one Fc variant pair dubbed “DEKK” consisted of substitutions L351D and L368E in one heavy-chain combined with L351K and T366K in the other drove efficient heterodimerization of the antibody heavy chains [27]. Additionally, using a common light chain, the bispecific antibody MCLA-128, targeting human EGF receptors 2 and 3, was produced and purified with a standard CHO cell culture platform and a routine purification protocol under Good Manufacturing Practice (GMP) conditions [27]. More recently, a full-length bispecific IgG-like bsAb was approved in 2017 was emicizumab (Hemlibra^®^) for the treatment of Hemophilia A patients [167]. Engineered on the structure of humanized IgG4, emicizumab mimics the function of activated FVIII to restore the FVIII binding to factor IX (FIX) and factor X (FX), which is missing in Hemophilia A patients [168,169]. Large-scale manufacturing of emicizumab was achieved by a combination of three antibody engineering strategies-a common light chain to assemble heavy and light chain, changing the charges of two different heavy chains to facilitate antibody purification, and the application of electrostatic steering of two different heavy chains to promote expression of heavy chains in cells [169]. Currently, numerous common light chain and common heavy-chain discovery platforms have been developed to enable the effective generation of antibodies for bsAb assembly. These include but are not limited to transgenic mice with a fixed single light chain [27] as well as screening phage display libraries with common heavy chain (as described above) [170,171,172]. Therefore, the application of a common light chain is becoming increasingly popular in this field in order to overcome the stability, yield, and immunogenicity problems of bispecific antibodies. However, this approach may lower flexibility in antibody engineering, which limits antibody optimization in some cases [173]. Furthermore, the screening process for common light chain requires animal immunization and/or phage display, which may be problematic due to time and development costs [166]. 

Different from the common light chain approach, CrossMab represents one of the most widely utilized generic approaches to solve the light-chain problem by exchanging the sequences of heavy and light-chain domains of Fab fragments. Crossmab technology allows for the generation of various bispecific antibodies including bi-, tri- and tetra-valent antibodies, as well as other novel Fab-based antibody derivatives [174]. Three different formats typically proposed are displayed in Figure 3b [142]. The first format involves simply replacing the entire Fab-arm of a heavy chain with a cognate light chain (CrossMab Fab) of one half of the bispecific antibody, and the “crossover” still retains the binding affinity while favoring the assembly of the engineered Fab fragment. The second format involves the swapping of VH of a Fab domain with its corresponding VL domain (CrossMab VH-VL) so that the molecular architectures of the heavy chain and light-chain interfaces in both arms of a bispecific antibody are not identical to prevent the light-chain mispairing. Likewise, for the third format, CH1 and CL of one arm of the bispecific antibody are also interchanged for the correct assembly between heavy and light chains (CrossMab CH1-CL) [142]. CrossMabCH1-CL yields no theoretical side products while CrossMabFab can result in the formation of a non-functional monovalent antibody due to the interaction between “VL-CL” of first IgG and “VH-CH1” of the second IgG [142,175]. CrossMabVH-VL can lead to the development of a Bence-Jones-like side product; to be more specific, successful CrossMabVH-VL should result in a pairing between “VH-CL” and “VL-CH1” while a Bence–Jones-like side product has a “VL-CL” chain paired with a crossed “VL-CH1” chain, meaning that two light-chain domains are assembled to one another. The Bence–Jones-like antibody can theoretically be prevented by making the two constant CH1 and CL electrostatically repulsive to one another [174]. 

The crossover design has been shown effective in target binding affinity and potency such as anti-tumor activity. Vanucizumab is one of the products that first utilized the CrossMabCH1-CL approach and was designed to target vascular endothelial growth factor (VEGF)-A and angiopoietin-2 (Ang-2) [142,176]. The design was optimized for clinical trial by using the original non-mutated bevacizumab targeting VEGF-A while applying the CrossMabCH1-CL mutation to LC06-bearing antigen-binding site targeting Ang-2. Furthermore, disulfide-stabilized knobs-into-holes mutations were introduced to ensure the correct heavy-chain assembly. As a result, vanucizumab exhibited high potency against patient-derived human tumors as well as several mouse tumors and was able to suppress micro-metastatic growth through Ang-2 inhibition without any side effect from anti-VEGF activity on physiologic vessels [176,177,178]. The technology has also been utilized to generate bispecific heterodimeric antibodies for many different purposes. For example, knobs-into-holes and CrossMabCH1-CL technology were used to produce a bispecific antibody targeting CD20 and HLA-DR as reported by Zhao et al. [146], and also to generate one of the broadest and potent HIV-1 neutralizing antibodies by Huang et al. [145]. The optimized CrossMab approach can be very effective and thus a powerful design strategy for improving selective light-chain pairing especially when used in combination with additional design approaches such as knobs-into-holes. The crossover design has been shown its effectiveness in achieving proper pairing of light chains for correct target binding affinity and consequently high yields. 

#### 2.2.4. Co-Culture Method

**Figure 4:**
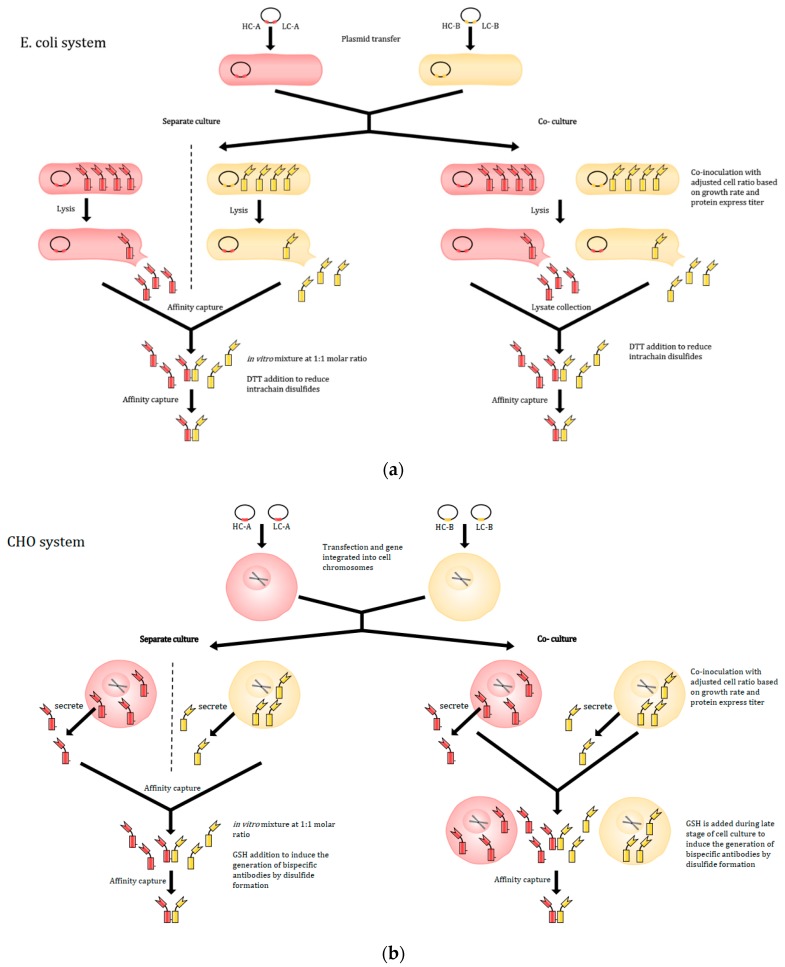
The illustration of the co-culture method to product IgG-like bispecific antibody in *E. coli* and CHO systems. (**a**) *E. coli* production vessels are constructed by transferring the cells with individual plasmids containing both heavy and light-chain gene for a half-antibodies. Cell lysis is used to harvest half-antibodies then assembled in vitro. Both separate culture and co-culture system can be used. (**b**) Similar to the *E. coli* system, CHO cells are co-transfected separate plasmids containing heavy or light-chain gene for a half-antibodies. Secreted antibodies are assembled by GSH induction. GSH: reduced glutathione.

Alternatively, to solve the light-chain mispairing issue and retain the natural antibody architecture, Spiess et al. proposed to produce bispecific antibodies by combining two distinct half-antibodies, expressed from two different cell lines in vitro [148] as depicted in Figure 4. Half-antibodies are then purified and mixed with 1:1 molar ratio in vitro to generate functional bispecific antibodies. While this half-antibody method can be effective, the method is attended with some inherent challenges. Using two separate cell lines means that two culture vessels, harvests, and purification processes must be performed before combining in vitro, potentially increasing costs and the risk of contamination [147]. The co-culture method was first demonstrated in *E. coli* in which cells were transformed with plasmids containing different half-antibodies genes (A and B) containing knobs-into-holes to prevent self-dimerization of the heavy chains prior to association with light chains [148]. After culturing, the cells are lysed to harvest half-antibodies. Since the processes mentioned above are identical for both cell lines, a co-culture strategy can be applied to lower the risk and cost. Both *E. coli* cell lines containing plasmid for half-antibody A and plasmid for half-antibody B are inoculated into the same vessel with same cell numbers. Using comparable cell numbers for both cell lines is a way to ensure having the same amount of antibodies A and B produced at the end. After culturing and processing, functional bispecific antibodies are successfully detected and harvested at the end. This method has been proven to be simple for the design and production of a wide range of stable antibodies [148].

Recently, the co-culture methodology has been shown to work for CHO cells as well [147]. Although similar in approach, there are some differences between the methods used, mainly in plasmids’ designs, inoculation, and harvest. For CHO cells, heavy chains and light chains are introduced into the cells on separate DNA plasmid to avoid non-cognate heavy chain and light-chain pairing and ensure a sufficient antibody titer. It is also critical to adjust the ratio of the two CHO cell lines for each half-antibody production based on antibody titers and cell growth rate to maximize production of half-antibodies A and B with a 1:1 molar ratio. Prior to harvest, reduced glutathione (GSH) was added in order to enhance bispecific antibody productivity, since CHO cells can still generate a minimum number of knob or hole homodimers of the desired half-antibody [143,160]. The addition of GSH as a reducing agent can help prevent the homodimerization of the half-antibodies and formation of disulfide. In the study, they also determined a 0.04 to 40 L scalable range for bispecific antibody production using co-culture [147]. With the simplicity of antibody design, relatively low risk and low cost, compatibility with current technology like controlled Fab-arm exchange (cFAE) and SEED, this methodology provides a simple and effective tool to produce a wide range of bispecific antibodies via co-culture of half-antibody secreting CHO cells. 

#### 2.2.5. Expression and Production of IgG-like Bispecific Antibodies 

Mammalian cells are the predominant workhorses for IgG production in industry, and the production platform is widely scalable for high titers of antibodies in order to meet clinical and commercial demands. However, the production of bispecific antibodies is more complex and typically requires at least two plasmids for heterodimerized heavy chains and one plasmid for a common light chain or two light-chain plasmids if two different light chains are used. Notably, expressing HC and LC on separate plasmids is recommended because the manipulation of the plasmid ratio is an easy and efficient approach to optimize protein assembly for desired products [179]. Subsequently, a laborious and time-consuming process is typically needed to select the most desirable clonal cell lines from a heterogeneous stable transfectant pool for large-scale antibody production [180]. CHO cell is well-known for its high protein productivity, low contamination rates, and human immunological compatibility [181]. The expression levels for antibodies via stable CHO cells can reach >3 g/L and sometimes >5 g/L and beyond and be successfully scaled up in bioreactors to large volumes [182]. Nevertheless, the yield of bispecific IgG-like antibody from CHO cells is lower about 1–3 g/L and often even lower [27]. 

Compared to stable transfections, transient transfection can deliver results in a few days without integrating recombinant DNA into the host genome. Human embryonic kidney (HEK293) and HEK-based Expi 293 cells are human cells for transient expression, which has been used early in bsAb development. However, due to the multiple number of plasmids required for the production of bispecific antibodies, transient expression of IgG can sometimes be difficult to scale and result in relatively low titers compared to stable cell lines. As an alternative, Rajendra et al. designed a single plasmid vector containing all the engineered light chains and heavy chains for both transient and stable expression of CHO cells. The CHO cell pools transiently transfected with two plasmid vectors of which one heavy chain and a light-chain pairing of the bispecific antibody were harbored on individual plasmids yielded 0.09–0.15 g/L with 73–92% correctly paired bispecific antibody as determined by mass spectrometry. The cell lines transiently transfected with a single plasmid vector containing all the components for bispecific antibody generated similar yields of correctly paired bispecific antibody [183]. However, a stable CHO pool with a single plasmid expression resulted in a higher titer ranging from 0.6 up to 2.2 g/L while the percentage of correct pairing ranged from 74 to 98% [183]. Their results indicated that a single plasmid system could be comparable to multiple plasmid system in terms of titers and it may facilitate the generation of stable CHO cells [183].

Indeed, for in vivo assembly, the efficient co-expression of engineered heavy and light chains relies deeply on the selection of stably expressing cell clones. Plus, culture conditions such as temperature also affect the half-antibody expression and aggregate formation [184]. In contrast, following downstream Protein A purification of CH3 mutants, in vitro assembly (separate expression of two different heavy-chain types followed by mixing them at appropriate redox conditions to induce assembly) have demonstrated their capability in generating high-quality molecules. Such is the case for Duobody technology [185]. With the Fab-arm exchange (FAE) process occurring in IgG4 in vivo and in vitro, researchers engineered FAE-associated IgG4-specific mutation pairs in IgG1 and generated stable IgG1 bispecific antibody with high yield and stability via in vitro assembly [186,187]. In addition, amenable bispecific antibody production can also be achieved by cell-free expression systems using *E. coli* based extracts. The flexibility of this system enables manipulation of the knob: hole plasmid ratio to achieve the most efficient HC heterodimer assembly, which can achieve protein yield at g/L scale within hours [143]. Nonetheless, these methods may include additional obstacles such as increased costs of production. As a result, co-expression in stable cells remains the predominant approach for IgG-like bispecific antibody production. 

## 3. Conclusions and Future Thoughts

This review has focused on the design, production, and quality of bispecific antibodies. A key challenge is how to produce uniform bispecific antibody with high quality and limited or negligible side products and impurities. For scFv-type bispecifics, the protein stability and tissue penetration ability vary and depend on different types of scFv antibody. Furthermore, with multiple host options to choose from, the determination of the most suitable system depends on the specific scFv antibody size, amino acid sequence, protein conformation, solubility, stability, purification, and scalability. For IgG-like full-size bispecific antibody, the production of pure heterodimer is achieved by complete heavy chain and light-chain heterodimerizations. Knobs-into holes method is an efficient means with which to associate different heavy chains. The common light chain and CrossMab technology are also useful approaches for varying light chain and heavy-chain assembly. More recently, co-culture and cell-free systems are also emerging as complementary production platforms to generate bispecific antibodies readily. 

Advanced protein and production engineering technologies in the antibody field have boosted the development of bispecific antibodies and their derivatives, which represent one of the fastest-growing next-generation of antibody therapeutics [188]. Diversity has been obtained in the bispecific antibody structure design both in the scFv- and IgG-like formats or by using a combination of both. Furthermore, the addition of small molecules such as aptamers, affibodies, and synthetic drugs can further expand their applicability, creating a plethora of novel bispecific antibody-related products [4]. Bispecific antibodies have found wide applicability to immunotherapy for cancer treatment, and these diverse molecules have the potential to treat other diseases, such as infections, acquired immune deficiency syndrome (AIDS) and genetic diseases [21] as well as serving for medical diagnosis purposes. Looking forward, with continuous efforts to improve their design, production, and purification on an industrial scale, bispecific antibodies will represent an increasing share of the therapeutics in the market with the capacity to reach their full potential as a complementary approach to the conventional therapy in the next decade.

## Figures and Tables

**Table 1:** Examples of the expression of bispecific antibody fragment molecules in various hosts.

Platform	Species	Molecule	Yield	Purification	Type	Reference
Bacteria	TG1 *E. coli*	anti-HER2/*neu* × anti-CD16	around 3.7 mg/L	Ni-NTA column	BiTE	[101]
Bacteria	*E. coli*	anti-TCR × anti-fluorescein	1 mg/L	Fluorescein affinity chromatography	Tandem bispecific scFv molecule linked by 212 and 205 c’ linkers	[67]
Bacteria	*E. coli, periplasmic*	anti-EpCAM × anti-CD3	12–15 mg/L	Ni-NTA column	BiTE	[102,103,104]
Bacteria	*E. coli BL21(DE3)*	anti-HER2 × anti-CD3	3 mg/L	Ni-NTA column	BiTE	[105]
Mammalian	CHO-K1	anti-CD123 × anti-CD3	2–5 mg/L	Protein G chromatography	BiTE-Fc	[106]
Mammalian	CHO cell	anti-P-cadherin × anti-CD3	1300 mg/L	Protein A chromatography	DART-Fc	[107]
Mammalian	CHO-S	anti-CD19 × anti-CD3		SEC	DART & BiTE	[40]
Mammalian	CHO	anti-CD33 × anti-CD3		IMAC + SEC	TandAbs	[48]
Mammalian	CHO	anti-CD19 × anti-CD3	>200 mg/L	Ni-NTA column	TandAbs	[71]
Mammalian	CHO cell	anti-EpCAM × ani-CD3		IMAC + gel filtration + CEX	BiTE	[108]
Mammalian	HEK 293	anti-EpCAM × ani-CD3		IMAC	BiTE	[109]
Mammalian	CHO-S	anti-GD2/DOTA-metal complex	5–10 mg/L	Protein A chromatography	IgG-ScFv	[110]

SEC: size exclusive chromatography, IMAC: immobilized metal affinity chromatography, CEX: cation exchange chromatography.

**Table 2:** Examples of the expression of IgG-like bispecific antibody molecules in various hosts.

Platform	Name	Target	Heavy-Chain Engineering	Heavy/Light-Chain Engineering	Yield	Purification	Note	Reference
CHO-DG44 cells	MCLA-128	Human epidermal growth factor receptors (HER2 and HER3)	knobs-into-holes	common light chain	0.6–1.2 g/L	Protein A + IEC	stable expression	[27]
HEK293F suspension cells	Ang-2-VEGF-A CrossMab	angiopoietin-2 (Ang-2) and vascular endothelial growth factor A (VEGF-A)	knobs-into-holes	CrossMab (CH1-CL)	0.03 g/L	Protein A + SEC	transient expression	[142]
cell-free system (*E. coli* extract)	ScFv-KiH, BiTE-KiH	CD3, EpCAM, HER2	knobs-into-holes		0.2–0.4 g/L	Protein A	in vitro	[143]
HEK293	M315-14D2 (scFv-Fc)	mouse NKG2D and mouse p55TNFR	Electrostatic Steering Effects		0.1 g/L	protein A	transient expression	[144]
Expi 293 cells *	10E8V2.0/iMab	human CD4 and HIV-1	knobs-into-holes	CrossMab (CH1-CL)		Protein A + SEC	transient expression	[145]
HEK293F suspension cells	CD20–243 CrossMab	CD20 and HLA-DR	knobs-into-holes	CrossMab (CH1-CL)		Protein A + SEC	transient expression	[146]
CHO-K1 suspension cell culture	anti-FGFR1/βKL	FGFR1/βKL	knobs-into-holes	Co-culture	0.35 g/L	Protein A + IEC	stable expression	[147]
*E. coli* K-12 W3110 suspension cell	Anti-Her2/CD3	Her2/CD3	knobs-into-holes	Co-culture	4.8 g/L	Protein A + HIC	stable expression	[148]
*E. coli* K-12 W3110 suspension cell	Anti-CD19/CD3	CD19/CD3	knobs-into-holes	Co-culture	1 g/L	Protein A + HIC	stable expression	[148]

* Expi293 cells are developed for high-yield transient expression purpose by Gibco company, which is based on suspension-adapted human embryonic kidney (HEK) cells. IEC: ion-exchange chromatography, SEC: size-exclusion chromatography, HIC: hydrophobic interaction chromatography.
